# Efficacy and Durability in Direct Labeling of Mesenchymal Stem Cells Using Ultrasmall Superparamagnetic Iron Oxide Nanoparticles with Organosilica, Dextran, and PEG Coatings

**DOI:** 10.3390/ma4040703

**Published:** 2011-04-07

**Authors:** Yi-Xiang J. Wang, Thibault Quercy-Jouvet, Hao-Hao Wang, Ak-Wai Li, Chun-Pong Chak, Shouhu Xuan, Lin Shi, De-Feng Wang, Siu-Fung Lee, Ping-Chung Leung, Clara B. S. Lau, Kwok-Pui Fung, Ken Cham-Fai Leung

**Affiliations:** 1 Department of Imaging and Interventional Radiology, Prince of Wales Hospital, The Chinese University of Hong Kong, Shatin, NT, Hong Kong, China; E-Mails: yixiang_wang@cuhk.edu.hk (Y.-X.J.W.); coldhot33@163.com (H.-H.W.); shilin@cuhk.edu.hk (L.S.); dfwang@cuhk.edu.hk (D.-F.W.); 2 Center of Novel Functional Molecules and Institute of Molecular Functional Materials, Department of Chemistry, The Chinese University of Hong Kong, Shatin, NT, Hong Kong, China; E-Mails: akwai0916@hotmail.com (A.-W.L.); sonicpong@cuhk.edu.hk (C.-P.C.); shhx@mail.ustc.edu.cn (S.X.); s0902552@mailserv.cuhk.edu.hk (S.-F.L.); Thibault-Quercy-Jouvet@enscp.fr (T.Q.-J.); 3 Institute of Chinese Medicine, The Chinese University of Hong Kong, Shatin, NT, Hong Kong, China; E-Mails: pingcleung@cuhk.edu.hk (P.-C.L.); kpfung@cuhk.edu.hk (K.-P.F.); claralau@cuhk.edu.hk (C.B.S.L.)

**Keywords:** magnetic nanoparticle, surface modification, cell labeling

## Abstract

We herein report a comparative study of mesenchymal stem cell (MSC) labeling using spherical superparamagnetic iron oxide (SPIO) nanoparticles containing different coatings, namely, organosilica, dextran, and poly(ethylene glycol) (PEG). These nanomaterials possess a similar SPIO core size of 6–7 nm. Together with their coatings, the overall sizes are 10–15 nm for all SPIO@SiO_2_, SPIO@dextran, and SPIO@PEG nanoparticles. These nanoparticles were investigated for their efficacies to be uptaken by rabbit bone marrow-derived MSCs without any transfecting agent. Experimentally, both SPIO@SiO_2_ and SPIO@PEG nanoparticles could be successfully uptaken by MSCs while the SPIO@dextran nanoparticles demonstrated limited labeling efficiency. The labeling durability of SPIO@SiO_2_ and SPIO@PEG nanoparticles in MSCs after three weeks of culture were compared by Prussian blue staining tests. SPIO@SiO_2_ nanoparticles demonstrated more blue staining than SPIO@PEG nanoparticles, rendering them better materials for MSCs labeling by direct uptake when durable intracellullar retention of SPIO is desired.

## 1. Introduction

Over the past two decades, our understanding in biology, materials science and nanotechnology has expanded rapidly. The inevitable intersection of these three disciplines has set in motion the development of an emerging research area, nanobiotechnology or nanobiomedical science, which offers exciting and abundant opportunities for discovering new processes and phenomena [1]. In particular, the advances in the synthesis and characterization of nanoscale materials allow scientists to understand and control the interactions between nanomaterials (e.g., nanowires, nanofibers, nanoparticles, nanobelts or nanoribbons, and nanotubes) and biological entities (e.g., nucleic acids, proteins, or cells) at molecular or cellular levels [2,3,4,5]. These advances promise major achievements in the life sciences.

By way of examples, superparamagnetic iron oxide (SPIO, Fe_3_O_4_) nanoparticles with appropriate surface functionalization can be used for numerous *in vitro* and *in vivo* applications, such as magnetic resonance imaging (MRI) contrast enhancement, cellular and molecular imaging, cell tracking, hyperthermia, targeted drug delivery, and cell separation [6]. All of these biomedical applications require that the nanoparticles possess high magnetization, uniform size, and a narrow particle size distribution [7,8,9,10,11,12]. Many of these applications also require peculiar surface coating and tunable magnetic properties of the magnetic particles [13], which are noncytotoxic, biocompatible, and also allow for a targeted delivery with particle localization in a specific area. Such magnetic nanoparticles can bind to drugs, proteins, enzymes, antibodies, or nucleotides and can be directed to an organ, tissue, or tumor using an external magnetic field [14]. Magnetic nanoparticles are usually coated with biocompatible layers such as dextran [15,16]. The SPIO@dextran or other nano/microparticles had been applied with ultrasonic wave [17] or applied with a relatively large amount of transfecting agent for effective cell labeling. However, transfecting agents such as lipofectamine are generally cytotoxic and relatively expensive, rendering them less preferred reagents. In this study, spherical, ultrasmall organosilica-coated (SPIO@SiO_2_), dextran-coated (SPIO@dextran), and polyethylene glycol (PEG)-coated (SPIO@PEG) nanoparticles were synthesized and utilized for direct labeling of mesenchymal stem cells (MSCs). Each type of particle was analyzed and characterized in order to control the level of functionalization and its efficiency for MRI relaxation enhancement. The direct uptake efficacies of these different nanoparticles by MSCs without any transfecting agent were studied.

## 2. Results and Discussion

MRI of SPIO-labeled cells has been proposed as an effective approach for non-invasive *in vivo* tracking of the localization and migration of targeted cells [18,19,20]. In some circumstances, the cells were labeled with SPIO particles *in vitro*, and then these cells were further administered to animal or human bodies so as to render them visible by MRI. Dextran-coated SPIO particles can be biodegraded by intracellular enzymes and acid [21]. Silica has been proposed as a coating material for SPIO core [22]. Silica has good biocompatility and hydrophilicity and can prevent the aggregation of particles in liquids and improve their physicochemical stability. For example, Bioglass which contains silica (~60 mol%), calcium, and phosphorus gives many applications in wound healing both in bone (hard) and soft tissues, middle ear implants, and in dentistry [23]. When organosilica is coated outside the SPIO core, the organosilica shell acts as a stabilizer, limiting the effect of the outside environment—intracellular enzymes and acids. The as-synthesized nanomaterials possess a similar SPIO core size of 6–7 nm. Together with their coatings, the overall sizes are 10–15 nm for all three types SPIO@SiO_2_, SPIO@dextran, and SPIO@PEG nanoparticles. These SPIOs are hydrophilic and dispersible in the cell culture media because of the presence of oxygen or nitrogen atom-rich tethers.

Hydroxyl functional SPIO nanoparticles are the precursors for coatings with organosilica, dextran, and PEG. Indeed, an inert organosilica coating on the surface of magnetite nanoparticles prevents their aggregation in liquid, improves their chemical stability, and provides better protection against cytotoxicity [24]. Moreover, by functionalizing the nanoparticles with –OH, –NH_2_ or –CO_2_H groups, the amine or carboxylic acid activated surface can be used to covalently link specific polymers, e.g., dextran and ethylene glycols. Dodecylbenzylsulfonic acid (DBS) was used as linker between SPIO and PEG. For potential medicinal and molecular imaging applications, surface functional nanoparticles are used for intravenous administration and so must be: (1) stable in aqueous solutions at physiological pH; (2) noncytotoxic and (3) remaining in the circulation for a long enough time to reach the target tissues.

**Figure 1:**
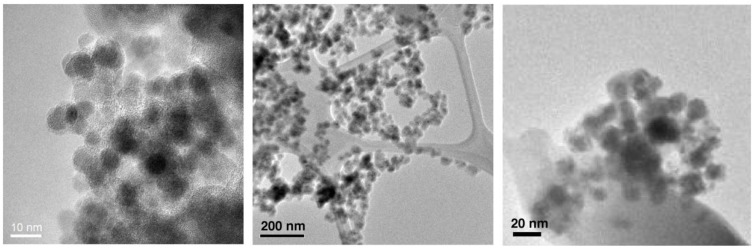
Transmission election microscopic (TEM) images of SPIO@SiO_2_ (Left), SPIO@dextran (Middle), and SPIO@PEG (Right) nanoparticles.

Transmission election microscopic (TEM) analysis clearly revealed (Figure 1) that each of the Fe_3_O_4_ nanoparticles (dark dots) possessed a coating layer (off-white layer), leading to a typical core/shell nanostructure. These nanomaterials possess a similar SPIO core size of 6–7 nm. Together with their coatings, the overall sizes are 10–15 nm for all SPIO@SiO_2_, SPIO@dextran, and SPIO@PEG nanoparticles.

In addition to the TEM characterization, inductively coupled plasma-optical emission spectroscopy (ICP-OES) and energy-dispersive X-ray (EDX) spectroscopy were employed for the determination of elemental contents (Fe, O, Si, C, *etc*.) of the nanoparticles (Table 1). The separate measurements by ICP-OES and EDX occurred with small errors that are less than 0.4%; there existed also small errors (<4%) when comparing the results obtained in both methods. Generally, ICP-OES is regarded as a more accurate method to determine the metal ion concentrations while EDX spectroscopy is an evaluation of the existence of elements in a specific area on TEM sample grid surface. The thickness of periphery coatings on SPIO would result in a decrease of iron percentage content. That is, comparatively, the thickness of coating is SPIO@dextran > SPIO@PEG > SPIO@SiO_2_. However, the difference of coating thickness is not significant for all three nanoparticle types, only *ca*. 5 nm by TEM analysis.

**Table 1:** Inductively coupled plasma-optical emission spectroscopy (ICP-OES) and energy-dispersive X-ray (EDX) results.

	ICP-OES	EDX			
	Fe%	Fe%	O%	Si%	C%
**SPIO**	63.9 ± 0.1	64.8 ± 0.2	35.2 ± 0.2	-	-
**SPIO@SiO_2_**	59.6 ± 0.1	57.1 ± 0.3	32.3 ± 0.3	3.5 ± 0.3	1.7 ± 0.3
**SPIO@dextran**	50.4 ± 0.1	48.8 ± 0.4	38.2 ± 0.3	-	3.1 ± 0.4
**SPIO@PEG**	53.0 ± 0.1	51.6 ± 0.3	37.4 ± 0.3	-	2.0 ± 0.4

Such small sized SPIOs were investigated for the feasibility to label MSCs, thereby observing the cells’ migration and homing by MRI. Clinically available SPIO such as Ferumoxide possess a relatively large diameter of *ca.* 180 nm [18,19]. As opposed to labeling the cells with larger but fewer SPIO particles, the development of nanosized (average particle diameter *ca.* 10–15 nm) SPIO nanoparticles may cause each individual stem cell to take up a larger number of SPIO nanoparticles than larger-sized SPIO nanoparticles. Subsequently, after cell proliferation, the nanoparticles possess enough numbers to be distributed into the offspring cells. The labeling of stem cells with a larger number of small SPIO nanoparticles will also be advantageous whereas exocytosis of SPIOs might occur after the initial labeling procedure. Additionally, there are data suggesting that small ionic particles are internalized into nonphagocytic cells with higher efficiency [25]. For ultrasmall SPIOs, a sufficiently strong magnetic performance must be ensured, and a SPIO particle with a core diameter of 5–10 nm seems to be ideal for such applications [6].

**Figure 2:**
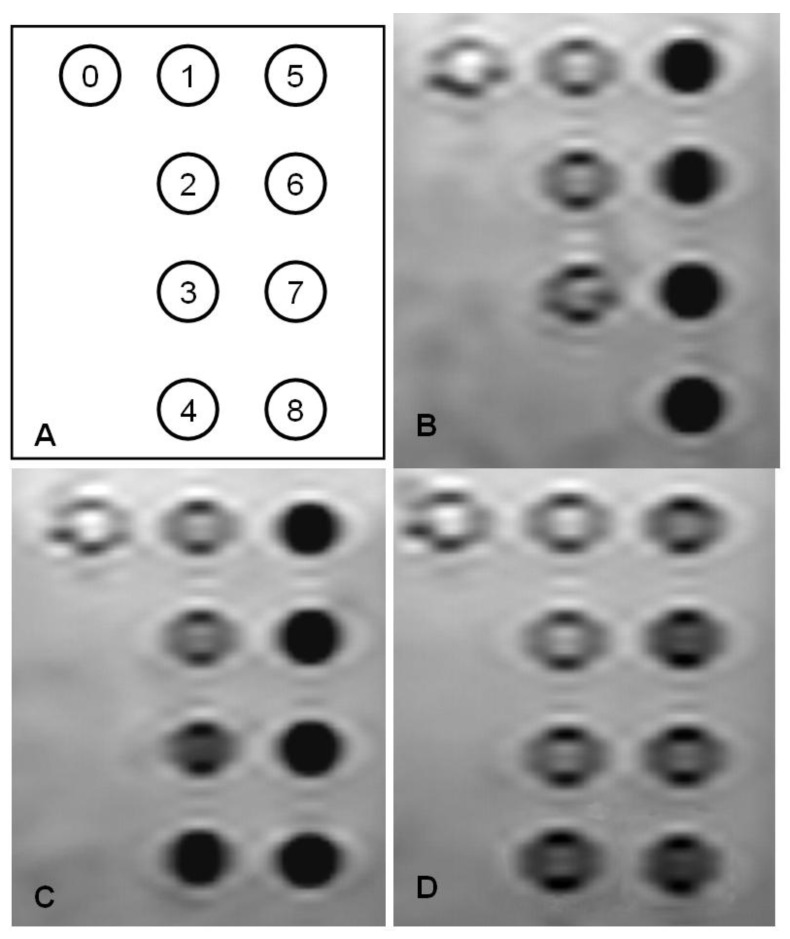
Spin echo MR image of the superparamagnetic iron oxide (SPIO) nanomaterials suspensions. **(A)** Diagram for iron concentration series; **(B)** SPIO@SiO_2_ **(C)** SPIO@dextran; **(D)** SPIO@PEG. The concentrations are (0): Deionised water, (1) 0.1 µgFe/mL; (2) 0.3 µgFe/mL; (3) 0.6 µgFe/mL; (4) 1 µgFe/mL, (5) 2 µgFe/mL; (6) 3 µgFe/mL; (7) 5 µgFe/mL; (8) 10 µgFe/mL. Note that the SPIO@SiO_2_ concentration of 1 µgFe/mL was not measured by MRI.

The *in vitro* MRI results are shown in Figure 2. With a spin echo sequence, the time of repetition (TR) = 2000 ms, and time of echo (TE) = 480 ms, signal attenuation can be visualized at 0.1 µgFe/mL for SPIO@SiO_2_ and SPIO@dextran, and 0.3 µgFe/mL for SPIO@PEG. The MRI relaxivity *r*_2_ was 60.8 ± 7.8, 79.3 ± 16.6, and 39.2 ± 10.7 mM^−1^ s^−1^ for SPIO@SiO_2_, SPIO@dextran, and SPIO@PEG, respectively. These *r*_2_ compared favorably with known SPIOs of similar size. VSOP-C184 (diameter 7 nm, Ferropharm, Teltow, Germany) possesses a *r*_2_ value of 33.4 mM^−1^ s^−1^, SHU-555C (diameter 21 nm, Schering, Berlin, Germany) possesses a *r*_2_ value of 38 mM^−1^ s^−1^, Sinerem (diameter 15–30 nm, Guerbet, France) possesses a *r*_2_ value of 65 mM^−1^ s^−1^ [18]. It is known that SPIOs of larger diameter can be associated with stronger *r*_2_ relaxivity. During *in vivo* application, SPIOs’ MRI relaxivity can be further enhanced with gradient echo sequence, longer TE, and higher magnetic field.

In this study, transfecting agent was not employed for MSC labeling. Transfecting agents are highly charged macromolecules that have been used to transfect oligonucleotides into cells via electrostatic interaction, which result in endosome formation [26,27,28,29]. Transfecting agents are cytotoxic whereas the toxic effect is proportional to the transfecting agent concentration [30]. Generally, an equal amount of transfecting agent was premixed with the nanomaterials before cell incubation. For rabbit MSCs labeling, in the absence of any transfecting agent, the labeling efficiency for MSCs with SPIO@dextran was rather limited and unable to reach the levels of using SPIO@SiO_2_ and SPIO@PEG. It has also been reported in literature that the most commonly used dextran-coated iron oxide nanoparticles do not present sufficient cellular uptake to enable cell tracking, probably because of a relatively inefficient fluid-phase endocytosis pathway [31].

**Figure 3:**
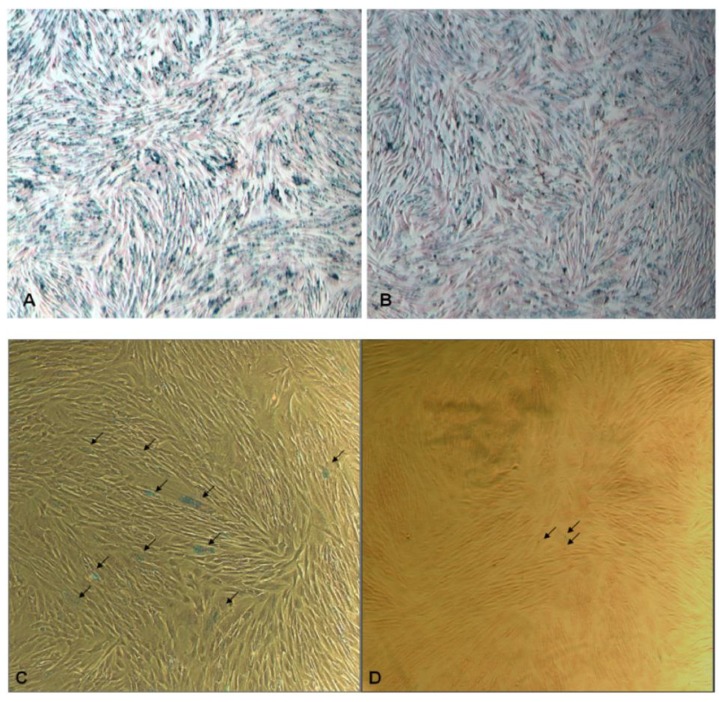
Optical microscopic images of **(A)** SPIO@SiO_2_-labeled and **(B)** SPIO@PEG-labeled mesenchymal stem cells (MSCs) post Prussian blue staining at day 0 (original magnification: ×20). The MSCs in **(A)** and **(B)** were extensively labeled with SPIO. The blueness values are *ca.* 14 for both figures. **(C)** SPIO@SiO_2_-labeled MSCs after three weeks (original magnification: ×40); there are multiple blue stained dots (arrows). **(D)** SPIO@PEG-labeled MSCs after three weeks (original magnification: ×40); it shows only trace amount of blue stained dots (arrows). The intensity of blueness for MSCs labeled with SPIO@SiO_2_ nanoparticles was 2.4 while that of MSCs labeled with SPIO@PEG nanoparticles was 0.0037.

Based on quantitative blueness measurements at day 0, MSCs with the same SPIO loading with SPIO@SiO_2_ or SPIO@PEG nanoparticles were selected for further culture and expansion. SPIO@SiO_2_ and SPIO@PEG labeled MSCs demonstrated normal cell grow and proliferation. The initial blueness measurement, which reflects iron loading within MSCs, was *ca.* 14 for MSCs labeled with both SPIO@SiO_2_ and SPIO@PEG nanoparticles (Figure 3A, B). The attenuation of the blueness measurement, reflecting loss of SPIO within MSCs, can be due to its biodegradation, as well as due to dilution of cell division and exocytosis [32]. On day 21 post SPIO labeling, it was observed that MSCs with SPIO@SiO_2_ rendered more blue staining spots than the MSCs-labeled with SPIO@PEG (Figure 3C, D). The quantitative blueness measurement for MSCs labeled with SPIO@SiO_2_ nanoparticles was 2.4 while that of MSCs labeled with SPIO@PEG nanoparticles was 0.0037. These results demonstrate that organosilica coated SPIO nanoparticles will be particularly useful for long-term labeling of cells that possess the potentials for further regeneration and division.

Recent experimental and clinical evidence demonstrated that stem cell therapy holds great potential for applications in a variety of clinical disciplines. Stem cells are characterized by numerous cell divisions (self-renewal) and differentiation into more than one terminally differentiated cell type. Stem cells provide a source from which fully differentiated, functional cells could be derived and give promising efficacy [33,34]. In addition to the differentiation of stem cells into matured functional cells, transplanted stem cells can secrete a variety of growth factors and cytokines, thereby enhancing cell survival after injury, and can facilitate the migration of resident cells to the site of injury and promote the repair activity. Following early experimental success, stem cell therapy has entered the stage of clinical trials with many encouraging results [35,36,37,38,39]. The development of stem cell based therapies requires an *in vivo* assessment of stem cell distribution to target organs (homing) and engrafting. MRI tracking of labeled cells is well suited for this task, and SPIO particles are considered to be the preferred material for magnetic labeling of cells. This is because (1) they provide a significant change in signal per unit of metal, in particular on *T*_2_*-weighted images; (2) they are composed of biodegradable iron, which is biocompatible and can thus be reused/recycled by cells by using normal biochemical pathways for iron metabolism; (3) they can be easily detected by light and electron microscopy [18,19]. After the intracellular labeling with clinically available, large dextran-coated SPIO particles, these SPIO particles can be biodegraded by intracellular enzymes and acid, and diluted by rapid cell division [15,16]. It has been reported that within a lysosome or endosome (*i.e*., low pH) environment, dextran coated ferromagnetic particles can become solubilized in a few days [15,40]. After biodegradation, iron oxide nanoparticles are turned into soluble ions, and their superparamagnetic nature and therefore MRI *r*_2_ relaxivity are lost. To reduce the biodegradation rate from intracellular enzymes and acid, a thin layer of organosilica can be coated around the iron oxide core. The silica shell can act as a stabilizer by limiting the effect of the outside environment on the core particles, so that the SPIO nanoparticles will remain intact and retain their superparamagnetic nature for a longer period of time. It has also been reported that to improve the labeling efficiency of stem cells that do not express high endocytosis capacity, amine peripherally functionalized on the silica surface of polyhedral SPIO nanoparticles lead to a higher labeling efficacy [4,17].

## 3. Experimental Section

*General Methods.* All chemicals were purchased from Aldrich or Acros unless otherwise stated. The chemicals were of reagent grade and used without further purification. Materials were obtained as stated: Ferric chloride hexahydrate (FeCl_3_·6H_2_O, >99.7%), ferrous sulfate heptahydrate (FeSO_4_·7H_2_O, >99.0%), sodium hydroxide (>98%), sodium chloride (>99.5%), anhydrous absolute ethanol, ammonia solution (20%), dodecylbenzylsulfonic acid (DBS, 70 wt% in 2-propanol), aminopropyltriethoxysilane (APTES, 99%), dextran (70 kDa), and polyethylene glycol (PEG, 6 kDa). Water was obtained from Barnstead ROpure system and was degassed using high purity nitrogen before use. All chemical reactions were performed under high purity nitrogen. Sonicator was operated at 35 kHz (Elma Tl-H-5) in water at ambient temperature. All measurements were performed in triplicate for at least two separate occasions.

*Preparation of SPIO Nanoparticles.* Spherical SPIO nanoparticles were prepared by a chemical co-precipitation method with two equivalents of ferric chloride and 1 equivalent of ferrous chloride with aqueous sodium hydroxide solution. The co-precipitation mixture was comprised of 0.17 mM Fe(II), and 0.33 mM Fe(III) and to the mixture was slowly added to 30 mM NaOH with shaking to yield the precipitate. The precipitate was separated by centrifugation and washes with deoxygenated water. The SPIO nanoparticles were modified by heating over 120 °C in water in an autoclave for 12 hrs to afford the SPIO nanoparticles 6–7 nm in size. The precipitate was separated by centrifugation and washed with deoxygenated water twice and anhydrous ethanol for four times, then vacuum dried at 50 °C overnight to afford the spherical Fe_3_O_4_ SPIO nanoparticles.

*Preparation of SPIO@SiO_2_ Nanoparticles.* SPIO@SiO_2_ nanoparticles were produced by a hydrolysis reaction on the surfaces of the SPIO nanoparticles using APTES. First, the SPIO nanoparticles were ultrasonically re-dispersed in a solution containing ethanol and water mixture. The pH value was adjusted to 9 with ammonia solution. APTES was added dropwise under vigorous stirring, and then heated to reflux for overnight. After that, the precipitate was separated by centrifugation and washes several times with water and ethanol to afford the SPIO@SiO_2_ nanoparticles of approximately 10–15 nm.

*Preparation of SPIO@PEG Nanoparticles.* The recrystallized SPIO nanoparticles were ultrasonically dispersed in ethanol prior to an addition of a solution of DBS in 2-propanol. This procedure afforded the SPIO-DBS nanoparticles. Subsequently, PEG with a molecular weight of 6 kDa was dissolved in ethanol and then added dropwise to the SPIO-DBS nanoparticle solution in one pot. The mixture was stirred overnight and a precipitate was obtained. The precipitate was separated by centrifugation and washed with ethanol to give the SPIO@PEG nanoparticles.

*Preparation of SPIO@dextran Nanoparticles.* Dextran with a molecular weight of 70 kDa was first dissolved in 2 M HCl solution and reflex for 1 h. This solution was then added dropwise to the ultrasonically dispersed, recrystallized SPIO nanoparticles in ethanol. The mixture was stirred for overnight and a precipitate was obtained. The precipitate was separated by centrifugation and washed with ethanol to give the SPIO@dextran nanoparticles.

*Physical Characterization of Nanoparticles.* Evaluation of the physical property of the nanoparticles was carried out using transmission electron microscope (TEM), energy-dispersive X-ray (EDX) spectroscopy and inductively coupled plasma-optical emission spectroscopy (ICP-OES). TEM surface analysis was performed using a FEI TecnaiF20 Field Emission Transmission Electron Microscope. One drop of sample dispersed in ethanol was added to the carbon-coated copper grid and was allowed to evaporate to dryness. Average particle size and size distribution were determined by counting approximately 50 particles. Built-in EDX spectroscopy was performed by locating a region (~20 nm × 20 nm) with substantial amount of materials for elemental analysis. ICP-OES was performed on Optima 4300 DV ICP-OES. Samples were dissolved in 2% HCl solution with a few drops of SnCl_2_ solution. Iron absorption was observed at 238.204 nm. The iron contents in a dispersed SPIO solution (in terms of g/mL) as well as in each organosilica-coated SPIO nanoparticle (in terms of %) were determined.

*Magnetic Resonance Relaxometry.* MR relaxometry of the nanoparticles was performed using a clinical 1.5 T whole body MR system (Siemens Sonata, Erlangen, Germany) in combination with a knee radio frequency coil for excitation and signal reception. Five SPIO nanoparticle samples were dispersed in distilled water at iron concentrations in a range from 0.1 to 10 μg/mL. For MR measurements, dispersed SPIO solutions at different concentrations were filled in each Eppendorf tube (1.5 mL). Sonication was applied for 10 min prior to MRI. *T*_2_ relaxation times were measured using a standard Carr-Purcell-Meiboom-Gill pulse sequence [time of repetition (TR) = 2000 ms, time of echo (TE) range = 30–960 msec, 32 echoes, field of view (FOV) = 134 × 67 mm^2^, matrix = 128 × 64, slice thickness = 5 mm, NEX = 3]. *T*_2_ relaxation times were calculated by a linear fit of the logarithmic ROI signal amplitudes versus TE. The *T*_2_ relaxivities (*r*_2_) were determined by a linear fit of the inverse relaxation times as a function of the iron concentrations used.

*Cell Labeling.* The animal study was approved by the institutional Animal Experiment Ethics Committee. A 20-week-old male New-Zealand white rabbit was used. Bone marrow was aspirated from rabbit iliac bone and washed with Dulbecco modified eagle medium (DMEM, Gibco 31,600) (bone marrow:DMEM = 1:4). The mixture was spinned. Then the fat debris and supernatant was removed. The pellet was resuspended with 10% FCS (Fetal Calf Serum, Gibco 16,140) in DMEM. The cell suspension was transferred to 75 cm^2^ tissue culture flask. The cell culture was incubated at 37 °C with 5% CO_2_. Half of the basal medium was refreshed after 4 days and all the culture medium was refreshed after another 3 days. The adherent MSCs were grown in colony. The cells can be subcultured into other culture flasks for cell expansion after 5–7 days.

The MSCs of passage four were used, and cultured at same density at 6-well plate. MSCs were labelled with SPIO by incubating with FCS free DMEM culture media containing the SPIO nanomaterial overnight (12 h). Each SPIO nanomaterial was tested with a range of nontoxic iron concentrations. Transfecting agent was not used in the study.

Prussian blue staining was used to assess the labeling efficiency (*i.e.*, iron loading) in MSCs. For this, cells were fixed with 2.5% glutaraldehyde (Sigma G4004), the cells were incubated with 1% potassium ferrocyanide (Sigma P3289) and 2% HCl for 10–15 min, then 1% neutral red (Sigma N8002) was added to stain nucleus. The degree of blueness was quantified using the following methodology [41]. A computer program was written in Matlab 7.1 (Mathworks Inc., Natick, MA, USA) to quantify the blueness of the stained images. For each pixel, as the color information consists of three components, namely red, blue and green, the relative blueness (RB) was calculated as the ratio between the blue component and the addition of the red and green components. Only the pixels with RB above a certain threshold were selected to be of interest. This threshold was determined to be 1.0 this study, due to the empirically tested differentiability of the background (low RB) and the stained regions (high RB). The total RB values of interested pixels normalized by the size of the image (%) was calculated and taken as the blueness measurement for the whole image. Because the background region has been discarded from the calculation of blueness measurement, the blueness measurement is capable of reflecting the degree of blueness only in the stained regions, regardless of the intensity and color in the background. Based on blueness measurement values staining at day 0, MSCs with similar iron loading of SPIO@SiO_2_ or SPIO@PEG were selected for further culture. These cells were cultured at 37 °C with 5% CO_2_ and normal divisions were permitted. The culture procedure would let the SPIO merely attached to the surface of cells, rather than truly labeled intracellularly, to fall-off the cells and washed away by the culture medium [42]. On day 21 with four passages after the SPIO labeling, Prussian blue staining and blueness measurements were performed again . The above cell labeling and blueness measurement experiments were repeated three times.

## 4. Conclusions

Cell assays with SPIO nanoparticles that contain different coatings, have been compared on direct labeling durability without the use of any transfecting agent. The results suggest that the nanoparticle with organosilica coating (SPIO@SiO_2_) offers higher labeling efficacy or better protection to its SPIO core compared to biodegradable dextran and PEG coatings when the nanomaterials are transfected in stem cells.
